# The features of technetium-99m-DTPA renal dynamic imaging after severe unilateral ureteral obstruction in adult rabbits

**DOI:** 10.1371/journal.pone.0237443

**Published:** 2020-08-19

**Authors:** Changyin Wang, Chun Gao, Wasili Maimaiti, Shun Li, Qisheng Yang, Linglong Jiang

**Affiliations:** 1 Department of Nuclear Medicine, Zhongnan Hospital of Wuhan University, Wuhan, Hubei, China; 2 Second Clinical Faculty, Medical School of Wuhan University, Wuhan, Hubei, China; 3 Emergency Center Surgical Department, Zhongnan Hospital of Wuhan University, Wuhan, Hubei, China; University of Sao Paulo Medical School, BRAZIL

## Abstract

**Background:**

It is controversial to evaluate the function of hydronephrotic kidneys by renal dynamic imaging (RDI). Our aim was to study the features of renal dynamic imaging (RDI) at different stages after unilateral ureteral obstruction (UUO) and to investigate a method that could be reasonably used to evaluate renal function and predict renal functional recoverability.

**Methods:**

We made UUO models using fifteen adult New Zealand white rabbits and systematically observed the changes in kidney morphology, blood flow, radiotracer distribution and function by RDI. We then compared the differences in terms of imaging features between different periods and analyzed the relationship between blood flow and function in obstructed kidneys.

**Results:**

1) Obstructed kidneys gradually became larger than preoperative kidneys and contralateral kidneys (*P*<0.05) and reached their peak size between days 42 and 56, after which they gradually got smaller in size. 2)The correlation between the blood perfusion of the obstructed kidney and the obstruction duration (*r* = 0.125, *P* = 0.045) was very weak. In the initial period of obstruction, the perfusion of the obstructed kidney significantly decreased, followed by a sharp rebound in later days, and then the perfusion declined again. The peak in blood perfusion was on day 7. 3) The uptake rate of the obstructed kidney drastically decreased in the early stage and became lower than that of the contralateral kidney and the kidney before the operation (*P*<0.05), after which uptake increased gradually; the peak was on day 28. After that, uptake gradually decreased. 4) The grading of the radiotracer distribution in obstructed kidneys was positively correlated with the obstruction duration (*r* = 0.975, *P* = 0.000), and a uniform renal distribution was an early feature of obstruction. 5) The blood perfusion of the obstructed kidney and its functioning frequently increased or decreased simultaneously, but sometimes there was also a mismatch. The peak of renal blood perfusion recovery occurred prior to the peak of renal function recovery.

**Conclusion:**

In different periods of severe UUO, the imaged features of obstructed kidneys were different. These features are beneficial for determining the degree of hydronephrosis and renal function and predicting renal functional recoverability.

## Introduction

Hydronephrosis is a common urinary tract disease caused by various reasons such as urinary tract stones, infections, trauma, ischemia, congenital ureteral stricture or tumors, et al [1–4]. Unilateral ureteral obstruction (UUO) caused by various factors is a common cause of unilateral hydronephrosis [1–4]. Renal dynamic imaging (RDI) is often used to evaluate the degree of hydronephrosis and renal function level [5–12] and plays an important role in the clinical management of hydronephrosis [11]. However, there has been controversy regarding the use of RDI to assess the function of the hydronephrotic kidney. Lee et al. [7] and Khalil et al. [13] believed that renal function after UUO declined. This opinion has generally been accepted. However, Provoost et al. [14] found that the function of the hydronephrotic kidney could maintain a normal level for a long time. Moon et al. [15], Inanir et al. [16] and Ozcan et al. [17] even observed a contradictory phenomenon, in which kidney function increased inversely. By using RDI data obtained from animal experiments that were completed in our department, we studied the renal imaging features of severe UUO and analyzed the characteristics of obstructive renal function changes. We newly discovered some imaging features of RDI in severe UUO, and some perspectives that are different from some literatures come into being with it, as reported below.

## Materials and methods

### Experimental animals and ureteral obstruction models

This study was approved by the Animal Care and Use Committee of the Wuhan University Center for Animal Experiments (AUP Number: 2013110). Experimental animals were provided by the Wuhan University Center for Animal Experimentation/ABSL-III Laboratory, including 15 male New Zealand white rabbits weighing 2.2–2.7 kg. The Animal Experiment Center of Wuhan University is a Biosafety Level 3 Animal Laboratory with a constant temperature and humidity negative pressure barrier environment. We handed over all experimental animals to this laboratory for feeding and management. Biological safety and animal welfare including shelter, food, water, feeding environment, etc. were guaranteed, and were subject to Institutional Animal Care and Use Committee (IACUC) and Institutional Biosafety Committee (IBC) supervision. All animals were healthy and had normal renal function. Experimental rabbits were fasted overnight preoperatively. We used an aqueous solution of 1% sodium pentobarbital to anesthetize the animals by intraperitoneal injection at a dosage of 30 mg/kg (sodium pentobarbital/animal body weight) and then generated the animal models of right ureteral obstruction according to the casing method [18, 19]. The kidney was examined by ultrasound at 24 hours after the operation, and the results showed that the renal collecting system was separated and hydronephrosis was formed, indicating that the model was successfully made. Postoperative ultrasound results did not show the separation of the renal collecting system, and no hydrops were formed in the renal pelvis, which was considered to be a failure to make the model, and then the animal was excluded from the experiment. In the first week after surgery, the state of the experimental animals was observed once a day, and medical iodophor was applied to the wound to disinfect it. After that, we observed the animal’s condition every 2 days and applied iodophor to the wound until the redness and swelling of the wound subsided and the wound healed normally. All animals had good wound healing and no infection. We anesthetized the experimental animals in the same way, and then injected air into the vein to euthanize the animals by air embolism. After that, we excised the kidneys and made further pathological specimens of the kidneys.

### Renal dynamic imaging

The scanning instrument was a model E. CAM single photon emission computerized tomography (SPECT) instrument from the Siemens Company, Germany, that was equipped with a low energy high resolution collimator. The ^99^Mo-^99m^Tc generator was a product of Atomic Hi-Tech Co., Ltd., Beijing, China. Diethylenetriamine pentaacetic acid (DTPA) was a product of Xinkesida Pharmaceutical Technology Co., Ltd., Beijing, China. In this study, RDI was performed in the baseline state. The rabbits were prohibited to eat protein-containing food for more than 12 hours, but they could drink water freely and eat fruits and vegetables before examination. At this time, the renal function of the animals was in the baseline state. Before examination, the animals were anesthetized by intraperitoneal injection with 1% sodium pentobarbital at a dosage of 20 mg/kg (sodium pentobarbital/animal body weight). When the animal gait was unstable or the reaction to pinching significantly weakened, we quickly immobilized the animal on a special thin board to prepare it for scanning. The radiotracer was ^99m^Tc-DTPA; the injection site was in the ear vein, and the injection was performed with the “bolus” injection method. The imaging conditions were as follows: for full (or empty) syringe imaging, matrix 256 × 256, acquisition magnification 1.00, acquisition time 60 seconds; for blood flow phase imaging, matrix 256 × 256, acquisition magnification 2.67, acquisition time 3 seconds/frame, and a total of 20 frame images were acquired; for renal function phase imaging, matrix 256×256, acquisition magnification 2.67, acquisition time 10 seconds/frame, and a total of 30 frames were acquired. To avoid the influence of the attenuation of radiation by the thin boards on the results, we placed all full (or empty) syringes and animals on a dedicated homogeneous board, which was used to immobilize and scan the animals.

### Parameters for evaluation of the kidneys

We observed the renal imaging features with RDI before and after severe UUO and the resulting changes in renal morphology, blood flow and function. Renal blood perfusion is classified into five levels: 1) normal perfusion, 2) mild reduction in perfusion, 3) moderate reduction in perfusion, 4) severe reduction in perfusion, and 5) no perfusion. The classification criteria can be found in S1 File and S1 Table. The radiotracer distribution in obstructed kidneys was classified into six levels: 1) normal renal distribution, 2) uniform renal distribution, 3) mild radiotracer reduction in the renal pelvis, 4) obvious radiotracer reduction in the renal pelvis, 5) no radiotracer distribution in the renal pelvis, and 6) no kidney image. The grading criteria can be found in S2 File and S2 Table. The early (1–2 min) uptake rate of the kidney was calculated to evaluate renal function; for a detailed explanation, see S3 File.

### Statistical analysis

Experimental data were processed and analyzed using SPSS software (version 22.0; IBM Corporation, Armonk, New York, USA). The comparison between the left and right renal long-diameter and the comparison of the right renal long-diameter before and after obstruction were performed by a paired t test. Comparisons between the left and right renal radiotracer uptake rates and comparisons of the right renal radiotracer uptake rate before and after obstruction were performed using a paired t test. The correlation between the grading of blood perfusion of obstructed kidneys and the obstruction duration and the correlation between the classification of the radiotracer distribution of obstructed kidneys and the obstruction duration were determined using the Gamma correlation test for double-ordered categorical variables. The test level was *α* = 0.05; *P*< 0.05 was considered to indicate a statistically significant difference or correlation.

## Results

### Size of the obstructed kidney

Table 1 shows that the long-diameter of the right kidney was slightly smaller than that of the left kidney before obstruction; after UUO, the right kidney gradually became enlarged. Between days 42 and 56, the right kidney had the largest long-diameter, and then the long-diameter gradually became reduced. The change in the right renal short-diameter after UUO was similar to that in the long-diameter. Therefore, the obstructed kidney underwent a process of first becoming enlarged and then gradually getting smaller in size.

**Table 1:** Changes in the renal long-diameter after right kidney UUO in experimental rabbits.

Obstruction time	n	Long-diameter (mm)		Change in the proportion of the right renal long-diameter (%)	Between the right and left kidneys		Between before and after the obstruction of the right kidney	
		Left kidney	Right kidney					
					*t*	*P*	*t*	*P*
Before obstruction	15	34.39±1.52	32.12±1.22	-	-5.894	<0.001	-	-
On day 1	15	35.45±2.51	36.26±4.07	12.82±11.35	1.002	0.333	4.349	0.001
On day 2	15	35.98±1.70	37.64±2.78	17.16±7.34	3.749	0.002	8.990	<0.001
On day 3	15	34.91±1.66	39.14±3.62	21.96±11.70	6.508	<0.001	7.409	<0.001
On day 4	15	35.15±1.60	40.80±2.50	27.01±6.39	10.507	<0.001	16.207	<0.001
On day 5	15	36.15±1.88	42.70±2.93	33.08±10.12	9.286	<0.001	13.387	<0.001
On day 6	15	35.78±2.33	41.34±2.48	28.82±8.79	11.559	<0.001	13.427	<0.001
On day 7	15	35.72±2.27	42.93±2.73	33.75±9.08	12.647	<0.001	15.160	<0.001
On day 14	14	35.59±1.60	46.30±4.82	44.23±16.10	9.766	<0.001	10.687	<0.001
On day 21	13	35.25±2.98	48.99±4.65	52.33±13.34	10.348	<0.001	14.057	<0.001
On day 28	13	35.25±2.06	53.32±7.46	65.55±19.78	9.735	<0.001	11.165	<0.001
On day 42	10	35.47±2.26	55.00±9.48	70.67±27.36	7.489	<0.001	7.862	<0.001
On day 56	10	37.32±3.30	55.11±11.84	70.98±34.47	4.860	0.001	6.263	<0.001
On day 68	9	35.73±1.28	52.71±9.84	64.81±30.37	5.613	0.001	6.277	<0.001
On day 82	9	34.97±1.87	51.24±11.26	59.85±33.40	4.859	0.001	5.271	0.001

Change in the proportion of the right renal long-diameter (%) = [(Right renal long-diameter after obstruction-Right renal long-diameter before obstruction)/ Right renal long-diameter before obstruction] ×100.

### Blood flow perfusion of the obstructed kidney

**Fig 1:**
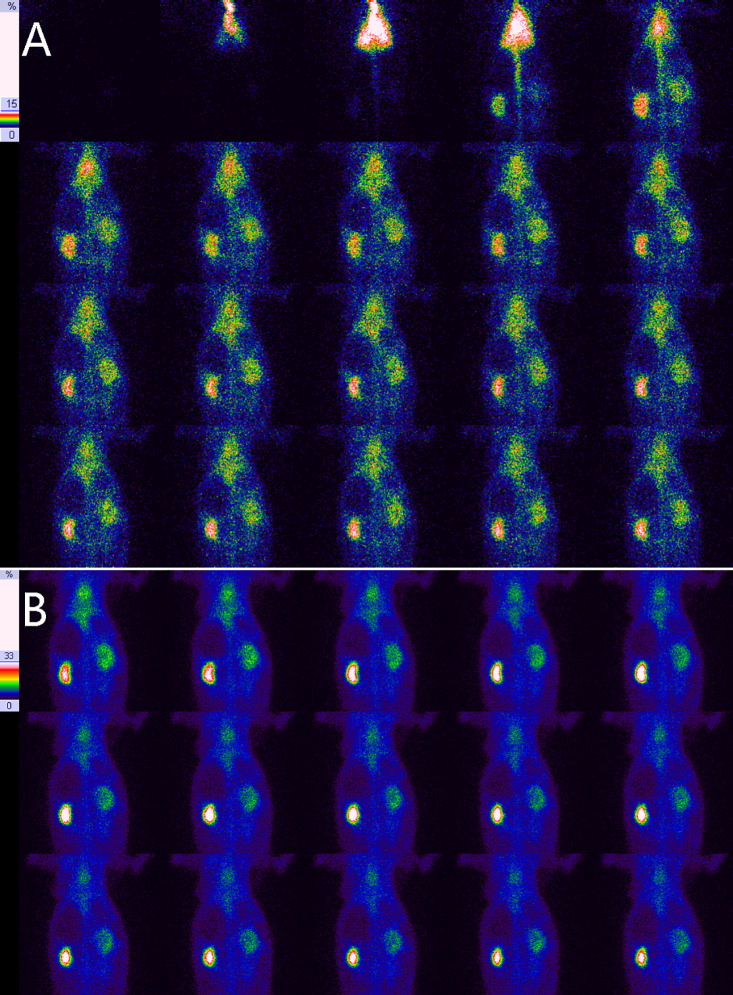
Results of RDI on the 3rd day for right UUO in experimental rabbits. **A**, Blood flow perfusion phase, showing a moderate reduction in right renal perfusion and that the peak of perfusion was delayed by 6–9 s in comparison with the left kidney. **B**, Renal function phase, showing a slight swelling in the right kidney and a normal distribution in the left kidney, in which the radioactivity level was gradually decreased from the center to the two poles; a uniform distribution is shown in the right kidney that is different from that of the left kidney.

**Fig 2:**
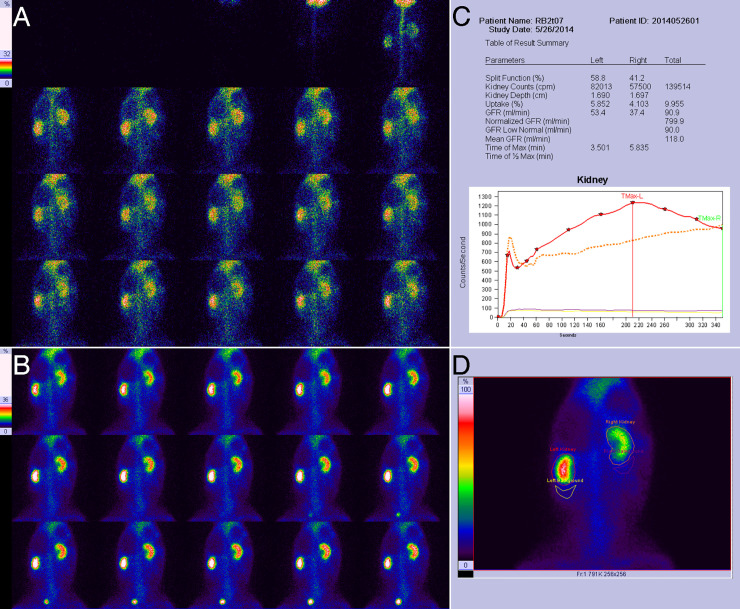
Results of RDI on the 7th day for right UUO in experimental rabbits. **A**, Blood flow perfusion phase, showing the normal perfusion of the right kidney, which is similar to that in the left kidney. **B**, Renal function phase, showing a mild radiotracer decrease in the right renal pelvis and mild swelling in the right kidney. **C**, Quantitative parameters and renogram curves. The left glomerular filtration rate (GFR) was 53.4 ml/min, the right GFR was 37.4 ml/min, and the right kidney curve presented a continuous rise. **D**, Drawing of the renal region of interest (ROI) and the background ROI.

**Fig 3:**
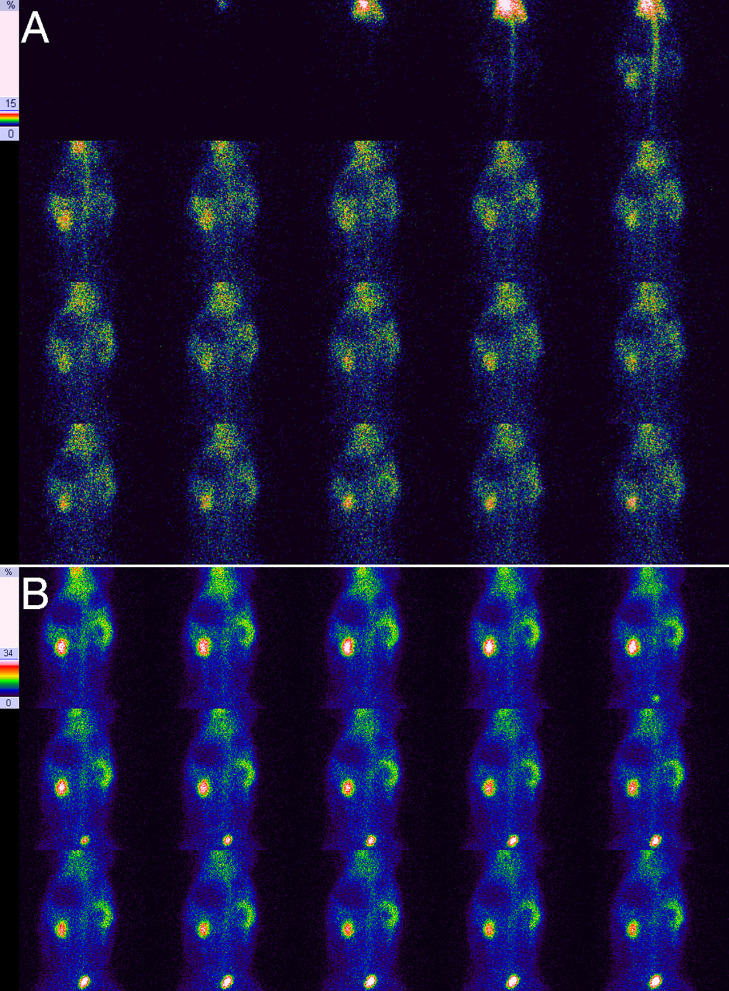
Results of RDI on the 21st day for right UUO in experimental rabbits. **A**, Blood flow perfusion phase, showing a severe reduction in right renal perfusion and that the peak in perfusion was delayed in comparison with the left kidney. **B**, Renal function phase, showing an obvious radiotracer decrease in the right renal pelvis and the swelling of the right kidney.

**Fig 4:**
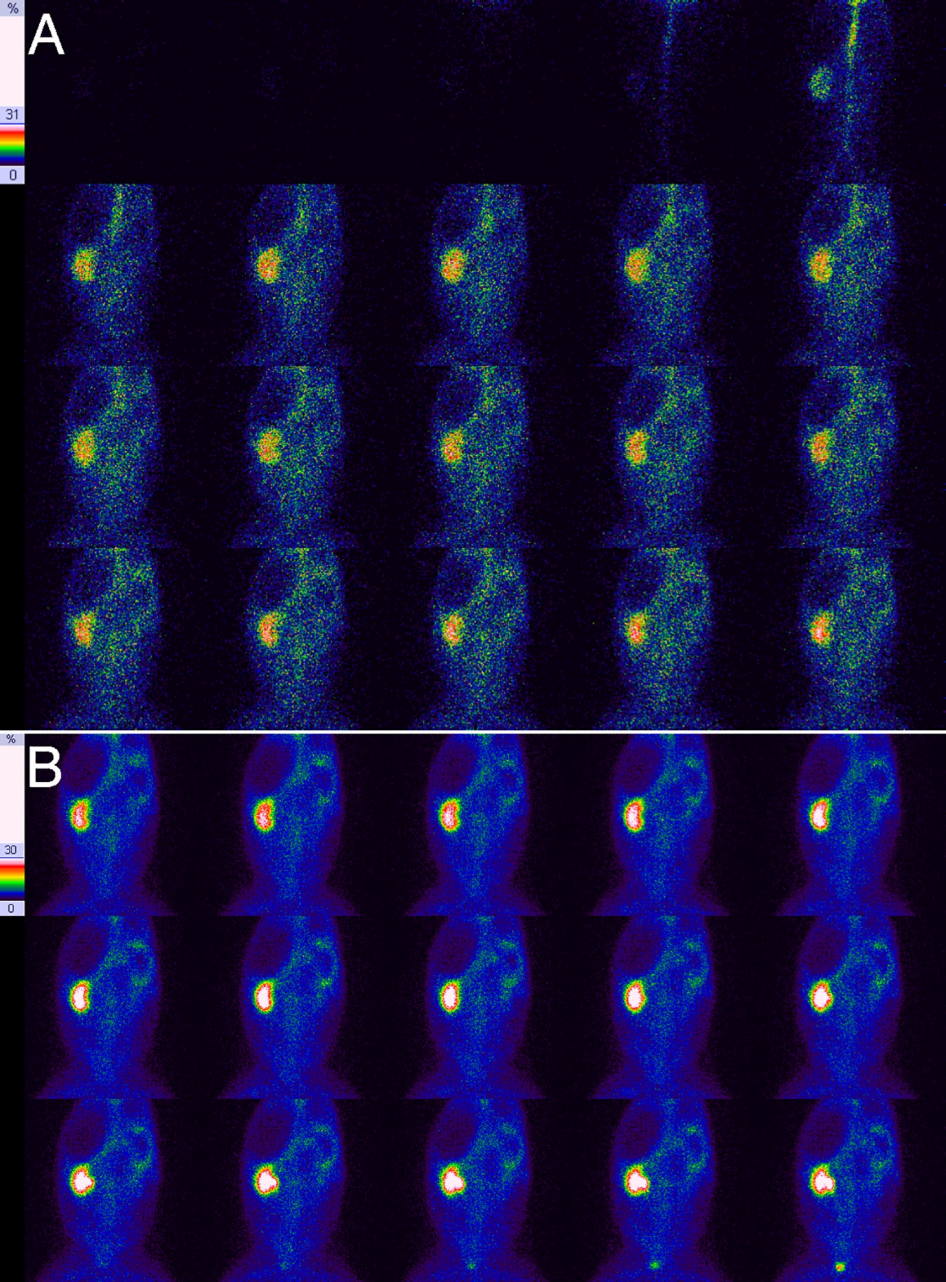
Results of RDI on the 69th day for right UUO in experimental rabbits. **A**, Blood flow perfusion phase, showing a lack of early perfusion in the right kidney and that the right renal perfusion was obviously slower and delayed in comparison with the left kidney. **B**, Renal function phase, showing the obvious swelling of the right kidney and a lack of radiotracer distribution in the right renal pelvis.

Table 2 shows the blood perfusion distribution in obstructed kidneys at different times during UUO. There was a weak positive correlation between the grading of blood perfusion of the obstructed kidney and the obstruction duration (*r* = 0.125, *P* = 0.045). Obviously, the blood perfusion of the obstructed kidney did not always decrease concomitantly with the obstruction time. Table 2 shows that the blood perfusion of the obstructed kidney was first universally decreased. In 75 examinations during the first 1–5 days after obstruction, there was no normal blood perfusion in any of the obstructed kidneys, and moderate to severe blood flow reduction was predominant (Fig 1). Afterwards, the blood perfusion of obstructed kidneys in some animals became increased to different degrees. On the 6th, 7th, and 14th day after obstruction, 27.3% (12/44) of examinations showed that the blood flow volume of obstructed kidneys had returned to normal levels (Fig 2). On the 7th day, the recovery rate was the greatest, reaching 40.0% (6/15). After 21 days of UUO, the blood flow volume of the obstructed kidney decreased again and universally presented a moderate to severe reduction(Fig 3) or even a complete lack of perfusion (Fig 4).Therefore, the blood flow perfusion of the obstructed kidney presented a process involving a large initial decline, a subsequent recovery and a return to normal levels, followed by a gradual decline again.

**Table 2:** Relationship between the grading of blood flow perfusion and the obstruction duration in the obstructed kidney after UUO (number of animals).

Grading of blood flow perfusion	Obstruction duration (days)													
	1	2	3	4	5	6	7	14	21	28	42	56	68	82
Normal perfusion	0	0	0	0	0	3	6	3	0	0	0	0	0	0
Mild reduction in perfusion	1	0	3	1	4	3	2	1	0	0	1*	1*	1*	1*
Moderate reduction in perfusion	9	4	1	8	5	3	1	4	4	9	6	5	4	2
Severe reduction in perfusion	5	9	7	6	4	2	6	4	7	4	1	4	2	2
No perfusion	0	2	4	0	2	4	0	2	2	0	2	0	2	4

*This was a special animal whose surgical incision was swollen and split after 28 days of obstruction. After 68 days of obstruction, the expanded ureter ruptured, and the obstruction was released naturally. As a result, the blood flow perfusion remained at a mildly reduced level on 42–82 days of obstruction. This was different from that observed in the other animals.

### Radiotracer distribution in the obstructed kidney

**Fig 5:**
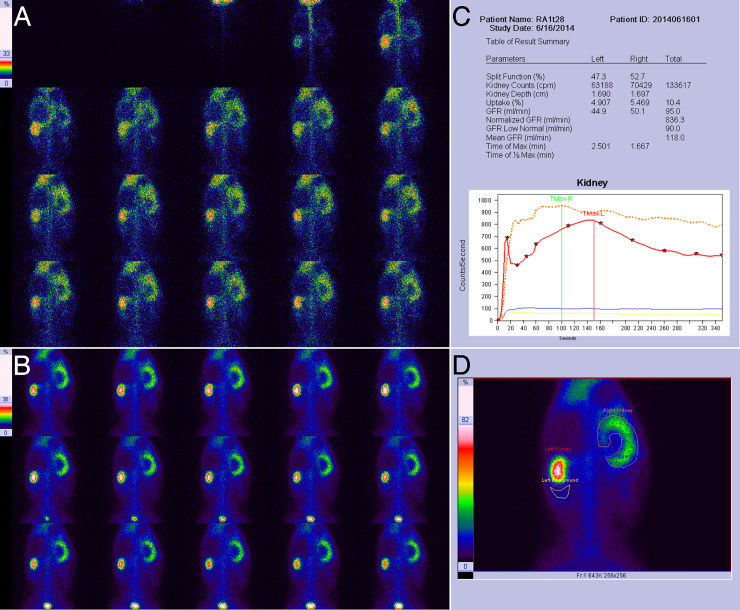
Results of RDI on the 28th day for right UUO in experimental rabbits. **A**, Blood flow perfusion phase, showing a moderate reduction in right renal perfusion and that the perfusion peak of the right kidney was delayed by approximately 6 s in comparison with left kidney. **B**, Renal function phase, showing a lack of radiotracer distribution in the right renal pelvis and obvious swelling in the right kidney. **C**, Quantitative parameters and renogram curves. The left GFR was 44.9 ml/min, the right GFR was 50.1 ml/min and the curve of the right kidney presents roughly a type of high-level extension line. **D**, Drawing of the renal ROI and the background ROI.

**Fig 6:**
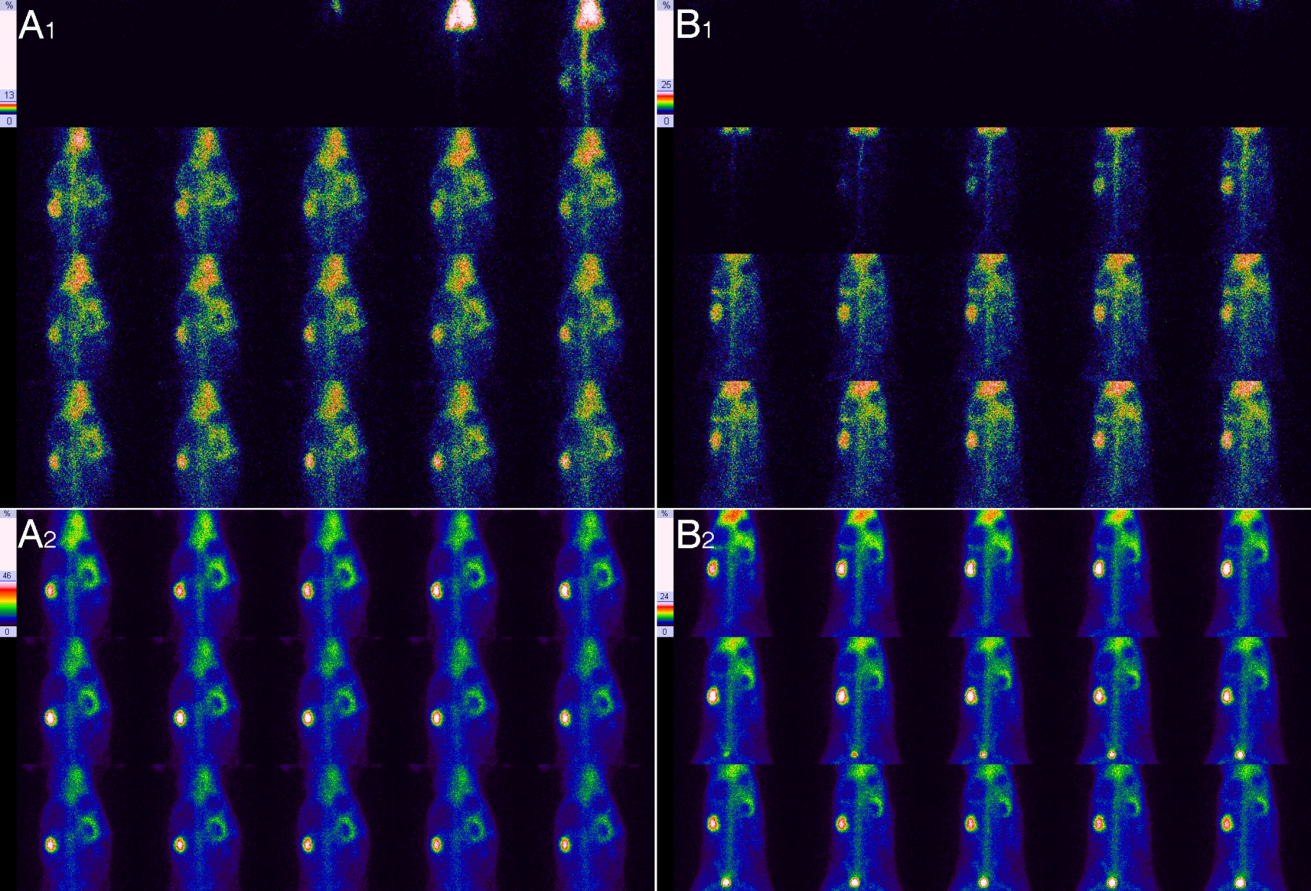
Comparison of the kidney size between the 28th and 82nd day of right UUO in the same rabbit. **A**, On the 28th of obstruction, **1** shows the blood flow perfusion phase, and **2** shows the renal function phase; the right renal area size was 1959.2mm^2^, the right renal long-diameter was 53.8 mm and the short-diameter was 39.8 mm. **B**, On the 82nd of obstruction, **1** shows the blood flow perfusion phase, and **2** shows the renal function phase; the right renal area size was 1044.9mm^2^, the right renal long-diameter was 38.7 mm and the short-diameter was 27.3 mm. The results show that the right kidney got smaller on the 82nd day in comparison with the 28th day of obstruction.

Table 3 shows the radiotracer distribution in the obstructed kidneys of experimental rabbits at different times. There was a strong positive correlation between the grading of the radiotracer distribution in the obstructed kidney and the obstruction duration (*r* = 0.975, *P* = 0.000). As shown in Table 3, all of the obstructed kidneys presented an abnormal distribution. During the first 1–5 days of UUO, 97.3% (73/75) of examinations did not show any visible renal pelvis, and they presented a uniform renal distribution (Fig 1). After that, the renal pelvis gradually presented a mild reduction in radiotracer (between days 5 and 28) (Fig 2), an obvious reduction in radiotracer (between days 21 and 28) (Fig 3) and no radiotracer distribution (between days 28 and 82) (Figs 4 and 5). On the 82nd day of UUO, the obstructed kidneys got smaller significantly (Fig 6), and their radiotracer distribution presented multiple variations, including a mild and obvious reduction in radiotracer in the renal pelvis, a lack of radiotracer distribution in the renal pelvis and even a lack of a renal image (Table 3). Obviously, uniform renal distribution after UUO is an early imaging feature. A reduction in radiotracer and a lack of radiotracer distribution in the renal pelvis are typical imaging features of hydronephrosis, while a lack of a kidney image is an imaging feature of late obstruction.

**Table 3:** Relationship between the grading of the renal radiotracer distribution and the obstruction duration after UUO (number of animals).

Grading of the renal radiotracer distribution	Obstruction duration(days)													
	1	2	3	4	5	6	7	14	21	28	42	56	68	82
Normal renal distribution	0	0	0	0	0	0	0	0	0	0	0	0	0	0
Uniform renal distribution	15	15	15	15	13	8	3	0	0	0	0	0	0	0
Mild radiotracer reduction in the renal pelvis	0	0	0	0	2	7	12	14	8	4	0	0	0	1
Obvious radiotracer reduction in the renal pelvis	0	0	0	0	0	0	0	0	5	8	0	0	0	2
No radiotracer distribution in the renal pelvis	0	0	0	0	0	0	0	0	0	1	10	10	7	4
No kidney image	0	0	0	0	0	0	0	0	0	0	0	0	2	2

### Early uptake rate of the kidney

Table 4 shows the results for the early uptake rate in kidneys in experimental rabbits. Before UUO, there was no significant difference in the radiotracer uptake rate between the bilateral kidneys (*P*>0.05). After UUO, the uptake rate of the obstructed kidney first decreased drastically and became lower than that of the preoperative kidney and the contralateral kidney (*P*<0.05). After that, the uptake rate gradually increased and peaked on the 28th day (Fig 5). At this time, there was no significant difference between the uptake rate of the obstructed kidney and that of the contralateral kidney or the preoperative ipsilateral kidney (*P*>0.05). After the peak occurred, the uptake rate of the obstructed kidneys decreased. Obviously, the functional parameters of the obstructed kidney showed a gradual process involving a significant decline in the early stage, then an increase to a level approaching that observed preoperatively, and finally a second decrease.

**Table 4:** Results for the early uptake rate (%) of kidneys in experimental rabbits.

Obstruction time	n	Uptake rate (%)		Between the right and left kidneys		Between before and after the obstruction of the right kidney	
		Left kidney	Right kidney				
				*t*	*P*	*t*	*P*
Before obstruction	15	5.37±0.81	5.25±0.85	1.78	0.097	-	-
On day 1	15	5.68±0.84	1.64±0.86	16.00	0.000	14.61	<0.001
On day 2	15	5.61±0.91	1.79±0.52	18.36	0.000	18.29	<0.001
On day 3	15	5.71±0.90	1.93±0.41	17.43	0.000	15.21	<0.001
On day 4	15	5.69±0.90	1.81±0.53	17.39	0.000	20.91	<0.001
On day 5	15	5.64±0.86	2.04±0.54	18.18	0.000	18.49	<0.001
On day 6	15	5.74±0.94	2.80±1.34	8.31	0.000	7.83	<0.001
On day 7	15	5.72±0.88	3.57±1.05	8.38	0.000	6.84	<0.001
On day 14	14	5.51±0.89	3.36±0.73	12.07	0.000	10.38	<0.001
On day 21	13	5.45±0.87	3.42±1.09	10.21	0.000	7.48	<0.001
On day 28	13	5.35±0.78	4.70±1.84	1.66	0.123	1.06	0.311
On day 42	10	5.43±0.90	2.26±1.18	11.72	0.000	8.66	<0.001
On day 56	10	5.67±0.90	1.95±0.92	19.52	0.000	17.82	<0.001
On day 68	9	5.43±0.95	1.90±1.36	8.08	0.000	7.69	<0.001
On day 82	9	5.57±0.85	1.91±1.37	9.11	0.000	9.69	<0.001

renal uptake rate (%) = [renal net radioactivity count rate (cpm) / net radioactivity count rate (cpm) of injected radiotracer] × 100.

## Discussion

### Imaging features of normal kidneys

After the radiotracer appears in the abdominal aorta, it will promptly appear simultaneously in the bilateral kidneys. The bilateral renal blood perfusion is similar and reaches a peak within 3 seconds after the abdominal aortic perfusion peak. The highest level of radioactivity in the bilateral kidneys was higher than that of the abdominal aorta. In the early renal parenchymal phase (1–3 min), the image of bilateral kidneys is very clear, and there is the highest radioactivity in the center of bilateral kidneys; the renal radioactivity gradually decreases from the renal center to the periphery. In the excretory phase, radioactivity in the urine gradually accumulates in the renal pelvis and drains into the bladder, which is accompanied by a gradual radioactivity decrease in the renal parenchyma. This describes the imaging characteristics of normal kidneys.

### Imaging features of obstructed kidneys

The obstructed kidney gradually accumulates urine, the renal pelvis expands, and the kidney swells. Renal blood perfusion in the early stage of obstruction is significantly reduced, which is similar to the ultrasound results obtained by Claudon et al. [20] and Hvistendahl et al. [21]. They believed that the increase in intrarenal pressure was an important cause of the decrease in renal blood flow [20, 21]. The early uptake rate of the obstructed kidney was also significantly reduced, indicating a significant decrease in filtration function. The radiotracer distribution first loses the normal feature of the highest radioactivity level being observed in the renal center and the lowest radioactivity level being observed in the renal periphery. The radioactivity in the renal center is relatively decreased, showing a uniform renal distribution from the center to the periphery. Therefore, the main features of early hydronephrosis after severe UUO are renal swelling, renal hypoperfusion, reduced renal filtration and a uniform renal distribution. Significant reduction in blood perfusion is a prominent feature in the beginning stage of severe UUO. A uniform renal distribution is a feature that is easily ignored; however, it is precisely the feature of early dilation of the renal pelvis. This feature of severe UUO has not been previously reported in the literature.

Then, the renal pelvis is further enlarged, and there is a decrease in or a lack of radiotracer distribution in the renal pelvis; the renal parenchyma becomes swollen or thinned, which is a typical imaging change in hydronephrosis. In the late stage of scanning, the radiotracer level in the renal parenchyma is gradually reduced, and the radioactivity level in the renal pelvis gradually increases, which is the most common clinical feature and is the diagnosis basis of hydronephrosis. However, this was rarely observed in this study. In contrast, the radiotracer level in renal parenchyma frequently increases with time, but the radiotracer does not obviously accumulate in the renal pelvis; the boundary between the renal parenchyma and the dilated renal pelvis is more distinct. This is different from the aforementioned commonly observed phenomenon, and the reason for this may be related to the degree of obstruction. In this study, the UUO was very severe, the intrarenal pressure was very high, the urinary flow was slow, and urine frequently stayed in the renal small capsule and renal tubules for a short period of time and thus was delayed in draining into the renal pelvis, which caused the renal parenchyma image to gradually become more clear and the boundary between renal parenchyma and renal pelvis to become more obvious. This is another imaging feature of severe UUO. As UUO progresses, the dilated renal pelvis begins to get smaller at a certain timepoint, and the significant decrease in renal function may result in no imaging of the renal parenchyma and decreased or no radiotracer distribution in the renal pelvis. The decreased or lack of radioactivity in the renal pelvis and the blurred or lack of imaging in the renal parenchyma is an imaging feature of the late period of severe UUO.

### Relationship between blood perfusion and renal function in the obstructed kidney

Our study shows that the blood perfusion of the obstructed kidney and its renal function presents a consistent trend of change. In the early and late stages of obstruction, blood flow and renal function generally decrease, which is similar to the result observed by Hvistendahl et al. [21]. However, Wåhlin et al. [22] believed that blood flow was increased in the renal expansion stage. We also found an increase in blood flow. In this study, the increase in blood flow did not occur in all renal expansion periods but mainly occurred within a small period of time. Both blood perfusion and filtration function in obstructed kidneys show their own recovery process, but the recovery peaks of renal blood flow and function are different. After an initial decline in renal blood flow and function parameters in UUO, the recovery peak of the blood flow often precedes that of the filtration function; the extent of the recovery of early blood perfusion is frequently higher than that of renal function, but in the middle stage of obstruction, the extent of the recovery of renal function is frequently higher than that of blood perfusion. Therefore, blood perfusion of the obstructed kidney sometimes does not correlate with renal function. Some scholars have found that renal blood perfusion gradually decreased along with the aggravation of obstruction or hydronephrosis [23–26], and thus, it was meaningful to indirectly evaluate glomerular filtration function according to blood flow perfusion. We found that the correlation between the blood flow perfusion and the function of the obstructed kidney is sometimes inconsistent, suggesting that using blood flow perfusion to assess the function of obstructed kidney is inaccurate.

### Can RDI accurately reflect the degree of UUO and renal function injury?

RDI can visually display renal pelvis expansion and the degree of hydronephrosis. Although renal images may have different characteristics in terms of the different degrees of UUO, RDI cannot accurately display the shape of the ureter and thus cannot directly reveal the degree of ureteral obstruction. Pyelectasis after obstruction is a gradual process, and the degree of hydronephrosis is related to the obstruction duration. In clinical practice, when we do not know the time of obstruction and the process of hydronephrosis, it is difficult for us to evaluate the degree of ureteral obstruction by the status and imaging characteristics of hydronephrosis. In this study, the degree of ureteral obstruction was the same in all rabbits, but the degree of hydronephrosis in RDI presented a large difference. The obstruction degree in the same animal was the same at different times, but the degree of hydronephrosis presented a great difference. Therefore, we cannot evaluate the ureteral obstruction degree according to the hydronephrosis degree or with RDI.

In this study, the baseline function of the obstructed kidney underwent a gradual change. Our animal models were made by using the same method as Cai Y et al. [18] and Ma SL et al. [27]. Ma SL et al. [27] believed that the histopathology of the obstructed kidney showed progressive damage. Therefore, the baseline function of the obstructed kidney is not consistent with its histopathological damage. The baseline function is not only related to its pathological damage but is also affected by intrarenal pressure [20, 21]. The initial decline in baseline function resulted from a significant increase in the intrarenal pressure. Later, the baseline renal function was greatly increased, which showed that the initial function change was characterized by an intrarenal pressure-dependent decline and also proved that the decline was recoverable. Obviously, the large fluctuation in the baseline function of the obstructed kidney and the inconsistency between its renal function and renal tissue damage show that it is difficult to accurately judge the degree of renal tissue damage and the true renal function based on baseline renal function parameters.

### Rational evaluation of obstructive renal function and the prediction of its recoverability

Li XD et al. [28] thought that it was not accurate to predict postoperative renal function recovery using preoperative renal function measurements. However, Kumar et al. [29] believed that patients with fewer changes in obstructive renal function before surgery showed better recoverability after surgery. We have a different opinion. In terms of the kidney morphology and radiotracer distribution according to RDI, our study showed that there were different imaging features at different stages of UUO, which is helpful for inferring the period of ureteral obstruction. In addition, the blood perfusion of the obstructed kidney was not always synchronized with the changes in renal function. At the peak time of blood flow, a decrease in kidney function that is not consistent with the blood flow change indicates that the function will be recoverable. In terms of the inconsistency of blood perfusion and function in obstructed kidneys, RDI also helps to identify the early stage of obstruction. Moreover, the degree of renal pathological damage is positively correlated with the obstruction duration [27, 30]. In the early stage of obstruction, the function of the obstructed kidney is less damaged and has significant recoverability, while the renal function in the late stage is severely impaired and the recoverability is weakened. Therefore, the judgment made during the obstruction period contributes to the accurate evaluation of renal function and the prediction of recoverability.

In fact, the judgment of renal function recoverability after UUO is affected by the inaccuracy of renal function measurement. In the early stage of obstruction, because the intrarenal pressure is increased, the measured renal function is inaccurate. The function of the obstructed kidney must therefore be recoverable; that is, the renal function can be recovered from its false appearance (measured value) to its original appearance (true value). In this study, the early function of the obstructed kidney showed significant recoverability without the release of the obstruction. If the obstruction is removed, renal function recovery is inevitable. In the late stage of obstruction, the morphologically getting smaller of the obstructed kidney is a manifestation of a significant decrease in renal function and indicates that the glomerular filtration rate is lower than the reabsorption rate of the renal tubules. Therefore, the intrarenal pressure gradually recovers, and the influence of the intrarenal capsule pressure on the measured value of the renal function is necessarily decreased. The measured value is close to the true value. At this time, the function of the obstructed kidney does not show significant recoverability.

## Conclusion

The animal studies showed that obstructed kidneys presented different imaging features at different periods of severe UUO. The results of this study are of great significance for guiding clinical practice. Clinically, the original state of the kidney before obstruction is often unknown. However, the morphology and function of bilateral kidneys are similar in normal individuals, so the unobstructed kidney can be used as a normal control for the obstructed kidney, which helps to improve the determination of the imaging characteristics of obstructed kidneys. The importance of RDI is to infer the UUO period in patients based on renal imaging features and especially to differentiate the early stage from the late stage of obstruction. On this basis, we can more reasonably assess the degree of renal impairment and predict the recoverability of renal function by combining the assessment of the quantitative parameters of renal function.

## Supporting information

S1 File Grading criteria for renal blood flow perfusion. (DOC)

S2 File Grading criteria for the renal radiotracer distribution. (DOC)

S3 File Why should the early uptake rate of the kidney be used to evaluate kidney function?. (DOC)

S4 File The original data set for supporting Table 1 in the main text. (XLSX)

S5 File The original data set for supporting Table 2 in the main text. (XLSX)

S6 File The original data set for supporting Table 3 in the main text. (XLSX)

S7 File The original data set for supporting Table 4 in the main text. (XLSX)

S1 Table Grading criteria for renal blood flow perfusion. (DOC)

S2 Table Grading criteria for the renal radiotracer distribution. (DOC)
