# Supplementary material for: The features of technetium-99m-DTPA renal dynamic imaging after severe unilateral ureteral obstruction in adult rabbits
Source: PLoS One. 2020 Aug 19;15(8):e0237443. doi: 10.1371/journal.pone.0237443 (PMC7437917; doi:10.1371/journal.pone.0237443)
Supplement: S1 File — (DOC) [file pone.0237443.s001.doc]

**S1 File**. **Grading criteria for renal blood flow perfusion**.

The images obtained within 15s after the radiotracer appeared in the abdominal aorta were selected to evaluate the blood flow perfusion of the kidney. By taking the contralateral healthy kidney and the abdominal aorta as a reference standard and observing the radioactivity level of the obstructed kidney after the radiotracer appeared in the abdominal aorta, the blood flow perfusion of the obstructed kidney was successively classified into 5 levels, from high to low, to evaluate the degree of blood flow reduction (S1 Table).
