# Supplementary material for: The features of technetium-99m-DTPA renal dynamic imaging after severe unilateral ureteral obstruction in adult rabbits
Source: PLoS One. 2020 Aug 19;15(8):e0237443. doi: 10.1371/journal.pone.0237443 (PMC7437917; doi:10.1371/journal.pone.0237443)
Supplement: S2 File — (DOC) [file pone.0237443.s002.doc]

**S2 File**. **Grading criteria for the renal radiotracer distribution**.

The uniformity of the renal radioactive distribution was evaluated by selecting images obtained 1-3 min after radiotracer injection. According to the uniformity of the renal radioactive distribution, the radioactivity density, the contour of the renal pelvis, the thickness of the renal parenchyma and the kidney size, and by taking the renal pelvis as the main observation point, the renal radiotracer distribution after UUO was classified into the following 6 levels for evaluating the degree of the radioactivity reduction in the renal pelvis (S2 Table).
