# Supplementary material for: The features of technetium-99m-DTPA renal dynamic imaging after severe unilateral ureteral obstruction in adult rabbits
Source: PLoS One. 2020 Aug 19;15(8):e0237443. doi: 10.1371/journal.pone.0237443 (PMC7437917; doi:10.1371/journal.pone.0237443)
Supplement: S3 File — (DOC) [file pone.0237443.s003.doc]

**S3 File**. **Why should the early uptake rate of the kidney be used to evaluate kidney function**?

The early uptake rate of the renal radiotracer is positively correlated with renal function, and it is the basis for the quantitative calculation of GFR by the Gates method [1-3]. In humans, the radiotracer ^99m^Tc-DTPA cannot enter the bladder for 2-3 min after injection. However, in some rabbits, the radiotracer can enter the bladder 2-3 min after injection, which is different from humans. Because the urine radiotracer level in the bladder cannot be distinguished as coming from the left or right kidney, the presence of urine radiotracer in the bladder will inevitably affect the accuracy of calculating GFR by the Gates method based on the 2-3 min renal uptake rate. Simultaneously, it also interferes with the accuracy of diagnosing kidney function by using the 2-3 min renal radiotracer uptake rate. For 1-2 min after injection, there is a very small amount of radiotracer entering the bladder in very few rabbits, and the radioactivity can be ignored. However, there is no radiotracer entering the bladder in most rabbits, so the 1-2 min renal radiotracer uptake rate is more favorable for accurately reflecting the split renal function. Therefore, we selected the image obtained 1-2 min after radiotracer injection to calculate the early uptake rate of the kidney to evaluate its glomerular filtration function.

**References**

1. Gates GF. Glomerular filtration rate: estimation from fractional renal accumulation of ^99m^Tc-DTPA (stannous). Am J Roentgenol. 1982; 138:565-70. <https://doi.org/10.2214/ajr.138.3.565>; PMID:7039273

2. Gates GF. Split renal function testing using Tc-99m DTPA: A rapid technique for determining differential glomerular filtration. Clin Nucl Med. 1983; 8:400-7. <https://doi.org/10.1097/00003072-198309000-00003>; PMID:6357589

3. Wang CY, Zhao YF. Influence of different outlining for region of interest in one kidney with Gates method on glomerular filtration rate of the contralateral kidney. Chin J Nucl Med Mol Imaging. 2016; 36:464-5. <http://www.chinadoi.cn/portal/mr.action?doi=10.3760/cma.j.issn.2095-2848.2016.05.019>
