# Supplementary material for: The features of technetium-99m-DTPA renal dynamic imaging after severe unilateral ureteral obstruction in adult rabbits
Source: PLoS One. 2020 Aug 19;15(8):e0237443. doi: 10.1371/journal.pone.0237443 (PMC7437917; doi:10.1371/journal.pone.0237443)
Supplement: S1 Table — (DOC) [file pone.0237443.s008.doc]

**S1 Table**. Grading criteria for renal blood flow perfusion

| Level | Blood flow perfusion | Imaging features |
| --- | --- | --- |
| 1 | Normal perfusion | The highest radioactive density of the obstructed kidney is higher than that of the abdominal aorta and similar to or slightly higher than that of the contralateral kidney. |
| 2 | Mild reduction in perfusion | The radioactivity density of the obstructed kidney is slightly lower than that of the contralateral kidney and is similar to that of the abdominal aorta. |
| 3 | Moderate reduction in perfusion | The radioactive density of the obstructed kidney is significantly lower than that of the contralateral kidney and lower than that of the abdominal aorta but is significantly higher than the background level; the edge of kidney is clear or roughly clear. |
| 4 | Severe reduction in perfusion | The radioactive density of the obstructed kidney is obviously lower than that of the contralateral kidney and abdominal aorta but higher than the background level. The imaging of the obstructed kidney is unclear and the edge is blurred. |
| 5 | No perfusion | The radioactive density of the obstructed kidney is slightly higher than the background or similar to the background, and the renal imaging is blurred, or there is no renal image. |
