# Supplementary material for: The features of technetium-99m-DTPA renal dynamic imaging after severe unilateral ureteral obstruction in adult rabbits
Source: PLoS One. 2020 Aug 19;15(8):e0237443. doi: 10.1371/journal.pone.0237443 (PMC7437917; doi:10.1371/journal.pone.0237443)
Supplement: S2 Table — (DOC) [file pone.0237443.s009.doc]

**S2 Table**. Grading criteria for the renal radiotracer distribution

| Level | Radiotracer distribution type of kidney | Imaging features |
| --- | --- | --- |
| 1 | Normal renal distribution | The highest radioactivity level is in the renal center, the renal radioactivity gradually decreases from the renal center to the upper and lower poles, and the renal pelvis outline is invisible. |
| 2 | Uniform renal distribution | The kidney is slightly swollen, and its radiotracer distribution is not characterized by a gradually decrease from the center to the two poles. The radioactivity level of the renal pelvis is similar to that of the surrounding renal tissues. The kidney presents a substantially uniform distribution, and the renal pelvis outline is unclear. |
| 3 | Mild radiotracer reduction in the renal pelvis | The kidney is mildly swollen, and the radioactivity of the renal pelvis is slightly lower than that of the renal parenchyma but significantly higher than that of the perirenal blood background. The outline of the renal pelvis is unclear. |
| 4 | Obvious radiotracer reduction in the renal pelvis | The kidney is enlarged, the renal parenchyma is swollen, the renal pelvis is dilated, and the radioactivity of the renal pelvis is significantly lower than that of the renal parenchyma but higher than that of the perirenal blood background. The renal pelvis outline is clear or substantially clear. |
| 5 | No radiotracer distribution in the renal pelvis | The renal parenchyma is visible, the kidney is obviously enlarged, the renal parenchyma is swollen and/or thinned, the renal pelvis is dilated, and the radioactivity of the renal pelvis is similar to or lower than that of the perirenal blood background. The outline of the renal pelvis is clear. |
| 6 | No kidney image | The renal parenchyma is invisible. The radioactivity of the renal parenchyma is similar to the perirenal background level. It is difficult to distinguish the renal parenchyma from the background. The radioactivity of the renal pelvis is lower than or equal to the perirenal background level. |
